# A Ceramic Rare Defect Amplification Method Based on TC-CycleGAN

**DOI:** 10.3390/s26020395

**Published:** 2026-01-07

**Authors:** Zhiqiang Zeng, Changying Dang, Zebing Ma, Jiansu Li, Zhonghua Li

**Affiliations:** 1 Engineering Training Center, Shanxi College of Technology, Shouzhou 036000, China; zzhqzhy@nuc.edu.cn; 2 School of Mechanical Engineering, North University of China, Taiyuan 030051, China; s202402049@st.nuc.edu.cn (Z.M.); ljs@nuc.edu.cn (J.L.); lzh2017@nuc.edu.cn (Z.L.)

**Keywords:** ceramic defect detection, image augmentation, generative adversarial networks, class imbalance

## Abstract

The ceramic defect detection technology based on deep learning suffers from the problems of scarce rare defect samples and class imbalance. However, the current deep generative image augmentation techniques are limited when applied to the task of augmenting rare ceramic defects due to issues such as uneven image brightness and insufficient features of small-sized defects, resulting in poor image quality and limited improvement in detection results. This paper proposes a ceramic rare defect image augmentation method based on TC-CycleGAN. TC-CycleGAN is based on the CycleGAN framework and optimizes the generator and discriminator structures to make them more suitable for ceramic defect features, thereby improving the quality of generated images. The generator is TC-UNet, which introduces the scSE and DehazeFormer modules on the basis of UNet, effectively enhancing the model’s ability to learn the subtle defect features on the ceramic surface; the discriminator is the TC-PatchGAN architecture, which replaces the original BatchNorm module with the ContraNorm module, effectively increasing the discriminator’s sensitivity to the representation of tiny ceramic defect features and enhancing the diversity of generated images. The image quality assessment experiments show that the method proposed in this paper significantly improves the quality of generated defective images. For the concave type images, the FID and KID values have decreased by 49% and 73%, respectively, while for the smoke stains type images, the FID and KID values have decreased by 57% and 63% respectively. The further defect detection experiments results show that when using the data set expanded by the method in this paper for training, the recognition accuracy of the detection model for rare defects has significantly improved. The detection accuracy of the concave and smoke stains types of defects has increased by 1.2% and 3.9% respectively.

## 1. Introduction

Since ancient times, China has been renowned as the “capital of porcelain”. Ceramic products such as tableware and vases have beautiful appearances, are easy to clean and safe, and are widely used in daily life. Surface defects such as cracks, bulges, black spots and dirt accumulation caused by insufficient production processes and unstable firing temperature during the ceramic production process have seriously affected the quality of the products [1]. Some high-end hotels and restaurants often have strict requirements for the quality of ceramics. Manufacturers need to screen out the defective ceramic products that do not meet the standards in advance. Compared with traditional manual inspection methods, establishing an intelligent defect detection system can significantly improve the detection efficiency and accuracy. However, the current deep learning-based defect detection technology in the field of ceramics faces a challenge of difficulty in constructing high-quality datasets, especially the problem of class imbalance caused by the insufficient number of rare defects [2]. Class imbalance can lead to prediction bias in the trained model. The model tends to classify the target as the defect category with a higher quantity, thereby reducing the model’s ability to identify the minority defect types. However, the commonly used image augmentation methods are limited by the uneven brightness of ceramic defect images and the scarcity of small defect features, making it difficult to generate high-quality defect images and having limited effect on improving the target detection performance. Therefore, it is urgent to study an effective and simple method for expanding ceramic defect images.

Currently, image augmentation methods [3] can be classified into image augmentation methods based on traditional image processing techniques and those based on model generation. Image augmentation methods based on traditional image processing techniques include geometric transformations (rotation, flipping, scaling, translation, cropping), color and brightness transformations, image erasing, image synthesis, image stitching, etc. Image augmentation methods based on model generation [4] generate completely new and realistic images by learning the data distribution patterns of the original images. Representative methods include Generative Adversarial Networks (GAN) [5], diffusion models [6], autoencoders [7], and variational autoencoders [8], etc. Among them, Generative Adversarial Networks (GAN) is an excellent generative model that can convert noise data into data that is close to the distribution of the training samples through training, and has been widely applied in image augmentation tasks in industrial defect detection.

Decourt C [9] proposed a generative adversarial network called DT-GAN, which can represent different defect types by learning background information. Experimental results show that the images generated by this method perform well in terms of fidelity and defect diversity, and still provide significant performance gains even with a small number of samples. Chen W et al. [10] developed an image generation model called DamperGAN based on multi-granularity conditional generative adversarial networks, which can generate high-resolution damper images and provide effective data support for the damper detection task. Ou H [11] proposed a ceramic defect detection data enhancement method based on StyleGAN3, which significantly reduces the data collection workload by generating ceramic defect samples. Experiments prove that this method has a short training time, a stable process, and helps improve the accuracy of the defect detection network. Yang B [12] proposed a new data enhancement algorithm called Mask2Defect, which first embeds prior knowledge into the teacher mask through the encoder, and then uses the generative adversarial network to convert the samples from the simulation domain to the real defect domain, thereby achieving controllable generation of defect attributes such as shape, severity, proportion, rotation angle, spatial position, and part number, and can generate a large number of diverse defect samples. Lv N [13] proposed D-sGAN for remote sensing image data enhancement, and its generator adopts the U-Net+ structure, and the discriminator is based on deep convolutional networks, which can efficiently generate high-quality change detection images and provide a data foundation for remote sensing image semantic understanding and other applications. Wang Y [14] proposed an FCGAN model, which integrates the attention mechanism into the generator and discriminator, and synthesizes high-quality defect images by recognizing the foreground area of the sample, which helps improve the accuracy of surface defect detection. Zhou [15] used a defect data enhancement method based on the improved pix2pix in the industrial product defect experiment. The generated defect images have a more realistic visual effect and a lower FID index, effectively improving the performance of the defect detection algorithm.

However, the pix2pix method relies on paired image data, which is often difficult to meet in actual industrial scenarios. Xia Y [16] proposed the RailGAN model, which enhances the generation effect by introducing a pre-sampling stage in CycleGAN. Experiments show that the generated images have a high similarity to the real images and are suitable for the training of neural network-based defect recognition algorithms. Chen K [17] adopted the sample enhancement strategy of Cycle-GAN to generate pipeline inner wall defect data to expand the dataset, thereby significantly improving the detection accuracy. Alam L et al. [18] improved CycleGAN by introducing perceptual image block similarity and structural similarity index to enhance the periodic consistency loss, resulting in significant improvements in peak signal-to-noise ratio (PSNR), universal image quality index (UQI), and visual information fidelity (VIF) indicators of the generated images.

In conclusion, CycleGAN possesses a powerful capability to perform cross-domain image transformation without requiring paired data. It can fully utilize the image information from defect-free samples, making it suitable for the task of expanding rare defects in ceramics. However, it is still limited by issues such as uneven brightness in ceramic defect images, blurred features of small targets, and significant size variations. Therefore, this paper proposes a new data augmentation method suitable for defect images of ceramics, named TC-CycleGAN (where “TC” is the Chinese pinyin abbreviation (Tao Ci) for “ceramics,” referring here to daily use ceramics). This method uses CycleGAN as the baseline model and improves the quality of generated defect images for daily use ceramics by optimizing the structures of the generator and discriminator.

## 2. Defect Image Augmentation Method Based on TC-CycleGAN

**Figure 1:**
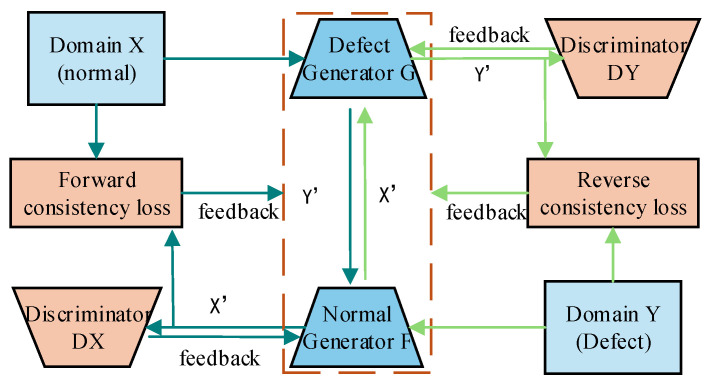
The basic principle of TC-CyceleGAN.

The method proposed in this paper, TC-CycleGAN, is a technique that alleviates the problem of class imbalance through image augmentation, thereby obtaining high-quality datasets and improving the accuracy of defect detection. Its structure is shown in Figure 1, which consists of Generator G, Generator F, Discriminator DY, and Discriminator DX. Domain X represents the set of normal images, and domain Y represents the set of target defect images. X and Y denote real images, while X′ and Y′ denote generated fake images. The generator takes source domain images and transforms them into target domain images. For example, generator G transforms received real normal images and generated normal images into fake target defect images, that is, generator G achieves $X\to Y^{\prime },X^{\prime }\to Y^{\prime }$; On the other hand, generator F transforms received real target defect images and generated target defect images into generated normal images, meaning $Y\to X^{\prime },Y^{\prime }\to X^{\prime }$. The discriminator is used to determine the authenticity of images within the corresponding domain. For example, DX discriminates whether the received normal image is real or generated by the generator, while DY determines whether the received target defect image is real or generated. During the training process, the model first fixes the generators to train the discriminators, then fixes the discriminators to train the generators. This alternating cycle continues until the discriminators can no longer effectively distinguish between generated images and real ones. At this point, the model reaches a Nash equilibrium, and the training concludes.

The cycle consistency loss is the core of CycleGAN, which ensures that the images can be reconstructed to be highly similar to the original image after forward and backward transformations, $\;X\to Y^{\prime }\to X^{\prime }\approx X,Y\to X^{\prime }\to Y^{\prime }\approx Y$. It effectively prevented the generator from experiencing excessive distortion during the conversion process, losing connection with the original image, and all source domain images being transformed into the same target domain image. This ensures the rationality and reliability of the image transformation process while effectively preventing model overfitting.

### 2.1. TC-UNet Generator

**Figure 2:**
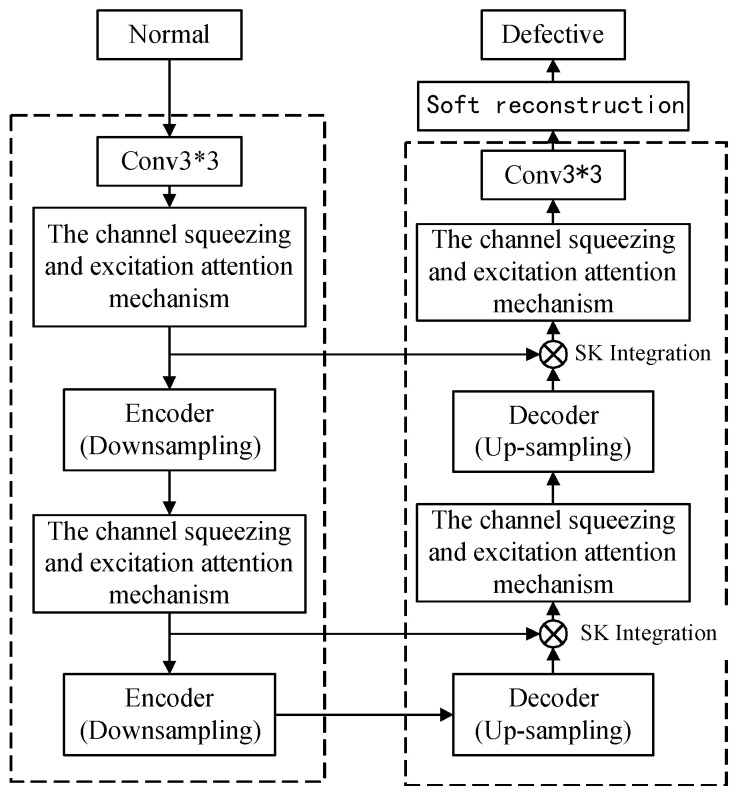
The Structure of TC-UNet Generator.

The surface of ceramic products is smooth but their shapes are irregular. Moreover, there is a large amount of dust in the production environment, which leads to problems such as uneven brightness and interference from dust in the captured images. Therefore, this paper proposes the TC-UNet generator, which takes UNet as the basic framework of the generator and optimizes it on this basis (Figure 2). It can effectively enhance the detail representation of the generated images and improve the overall quality of the generated images.

**Figure 3:**
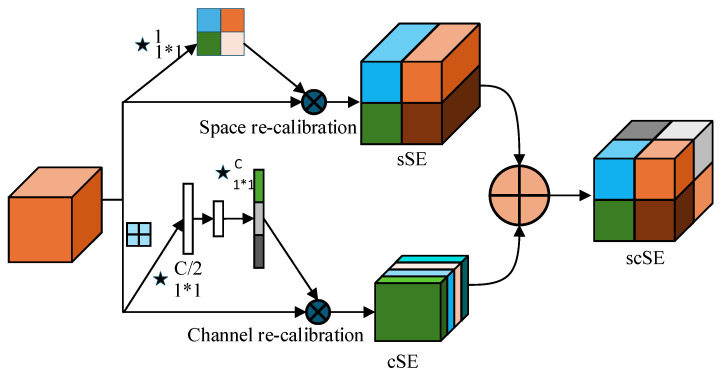
Spatial channel squeezing and stimulating attention.

This section first adds scSE [19] to the generator, as shown in Figure 3, scSE consists of cSE (Channel Squeeze-and-Excitation) and sSE (Spatial Squeeze-and-Excitation). This module can simultaneously adjust the feature map in both the spatial and channel dimensions, enhancing the network’s ability to focus on the details and spatial structure information of defects. With the help of the scSE module, the generator can more accurately focus on the defect area, effectively capture the shape and fine features of surface defects, while suppressing background noise and other disturbances, thereby significantly improving the quality of the generated image, and improving its clarity, realism, as well as the visual accuracy and detail representation of the defect area.

Secondly, a DehazeFormer module [20] was incorporated into the generator to enhance the model’s reconstruction performance on defect areas of ceramic surfaces, particularly during the reconstruction of point-like regions. The core of the DehazeFormer module lies in the SK fusion layer and the soft reconstruction layer, as detailed in Figure 2, both of which are located in the decoding stage. The SK fusion layer replaces the concatenated fusion layer, enabling the model to more flexibly integrate multi-scale defect information and better capture multi-scale defect features in ceramic surface defect images. The soft reconstruction layer replaces global residual learning, further strengthening the model’s adaptability to complex scenes and making the reconstruction results more realistic and clearer.

### 2.2. TC-PatchGAN Discriminator

**Figure 4:**
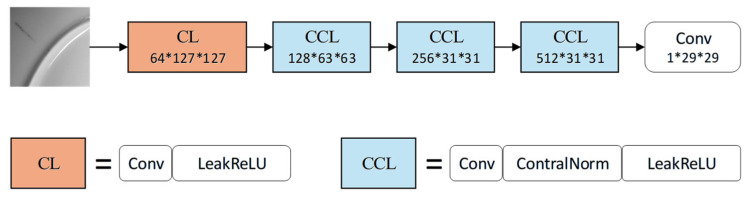
The structure of TC-PatchGAN Discriminator.

After the generator successfully generates the simulation images, these images will be input into the discriminator for quality assessment and feature analysis. This section proposes the TC-PatchGAN discriminator, which is constructed based on the PatchGAN architecture [21] and further improved to enhance its discrimination accuracy and computational efficiency. The optimized discriminator structure is shown in Figure 4.

PatchGAN decomposes the input image layer by layer into multiple overlapping local blocks, and independently performs authenticity discrimination within each block, assigning a probability value to each image patch that represents the likelihood of it being a real image. This local-region-based discrimination mechanism enables PatchGAN to finely capture the subtle features and texture information in the image, thereby effectively ensuring that the generated images have a high degree of visual realism in terms of visual details. Meanwhile, the PatchGAN encourages the model to generate realistic local textures rather than global, dataset-specific noise, effectively preventing model overfitting. To further enhance the sensitivity and discrimination ability of the discriminator in feature representation, and to improve the quality and diversity of the generated image samples, this section replaces the original BatchNorm layer with the ContraNorm module [22]. The BatchNorm layer is widely used in traditional PatchGAN discriminators, which can stabilize the training process and promote model convergence. However, as the network depth increases, BatchNorm may cause the dimension collapse problem, resulting in homogenized feature representations and thereby limiting the diversity of generated images. The ContraNorm module can significantly improve the uniformity and diversity of feature representations without introducing additional parameters, effectively alleviating the dimension collapse phenomenon. It not only helps maintain the diversity of feature distributions but also strengthens the model’s ability to perceive and extract subtle features. The calculation formula of the ContraNorm module is as follows:

(1) $$H_{t}=\left(1+s\right)H_{b}-s\left(H_{b}H_{b}^{T}\right)H_{b}$$

In the formula: $H_{t}$ represents the updated representation matrix; $H_{b}$ represents the representation matrix before update; s is the scaling factor, which is used to control the intensity of the update.

In the final stage, the generator receives the probability estimates from the discriminator and adjusts its own parameters based on this to optimize the quality of the generated images. Through the adversarial training between the generator and the Patch-based discriminator, a dynamic game is formed: the generator continuously optimizes its output to deceive the discriminator, while the discriminator continuously improves its ability to distinguish between real and fake images. During this iterative process, the generator gradually learns more complex and detailed image features, thereby making the generated images visually closer to the quality of real images. As the training progresses further, the generator not only can synthesize images with a high degree of realism, but also maintains consistency in style and structure with the target image, thereby effectively improving the performance of the conversion and generation tasks between normal samples and rare defect samples.

### 2.3. Loss Function

The CycleGAN loss function is mainly divided into two parts: the adversarial loss and the cycle consistency loss. The adversarial loss function is used to measure the distance between the generated samples and the real samples, and its mathematical expression is as follows:

(2) $$\begin{array}{c} L_{cyc}(D,Y)=E_{y∼p_{data}(y)}[{logD}_{Y}(y)]+E_{x∼p_{data}(x)}[\log(1-D_{Y}(G(x))] \end{array}$$

In the formula: X represents the data distribution of the entire source domain images, and Y represents the data distribution of the entire target domain images; x represents a source domain image, and y represents a target domain image. G denotes the generator, which transforms an image from the source domain to the target domain, expressed as G(x); D denotes the discriminator, which determines whether a generated sample is real or fake, where DY is the target domain discriminator that distinguishes whether a target domain image sample is real or fake; $p_{\mathrm{data}}\left(x\right)$ represents the probability density function of the real source domain sample data distribution, and $p_{\mathrm{data}}\left(y\right)$ represents the probability density function of the target domain sample data distribution; E denotes the expected value over real samples from either the source or target domain.

The cycle consistency loss refers to the distance between the transformed samples from the source domain to the target domain and then back to the source domain and the original samples. The mathematical expression of the cycle consistency loss function is as follows:

(3) $$\begin{array}{c} L_{cyc}(G,F)=E_{x∼p_{data}(x)}\left[∥F\left(G(x)\right)-x{∥}_{1}\right]+E_{y∼p_{data}(y)}\left[∥G\left(F(y)\right)-y{∥}_{1}\right] \end{array}$$

Among them, x represents an image from the source domain, and y represents an image from the target domain. G denotes the forward generator, which transforms a sample from the source domain to the target domain, expressed as G(x). F denotes the inverse generator, which transforms a sample from the target domain to the source domain, expressed as F(y). $∥\cdot {∥}_{1}$ represents the L1 norm, which calculates the absolute error between the generated sample and the original sample. F(G(x)) indicates that the source domain sample x undergoes transformations by the forward generator G and the inverse generator F to achieve the reconstruction of the source domain image x. G(F(y)) indicates that the target domain sample y undergoes transformations by the inverse generator F and the forward generator G to achieve the reconstruction of the image y. Other variables are the same as those in Equation (2).

## 3. Experimental Study on Image Amplification of Rare Defects in Ceramics

In this section, TC-CycleGAN is used to convert normal and defect-free ceramic images into rare ceramic defects. At the same time, experiments on quantitative analysis of image quality and verification of the effectiveness of target detection are conducted using FID (Fréchet Inception Distance) [23] and KID (Kernel Inception Distance) [24] as evaluation metrics, comprehendsively assessing the effectiveness of the proposed method in this paper.

### 3.1. Experiment Settings

The experiment was conducted under the Windows 11 operating system, with the CUDA 11.1 version of the parallel computing framework installed. The deep learning framework used was PyTorch 1.9.0. The processor was the i5-12600kf, and the graphics card was the NVIDIA GeForce RTX3060. The size of the input images was 512 × 512 pixels. The number of training rounds was 300, with each batch size set to 4. The optimizer was Adam, and the initial learning rate was 0.0002.

### 3.2. Data Set and Experimental Procedure

**Figure 5:**
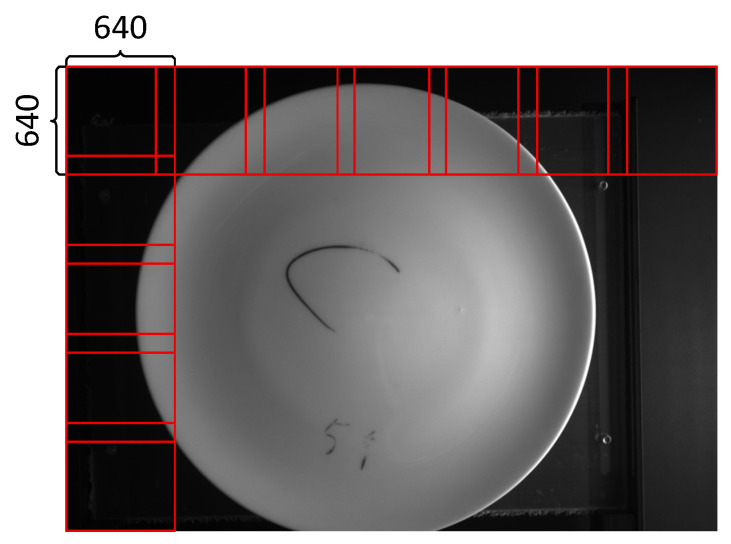
Clipping strategy.

**Figure 6:**
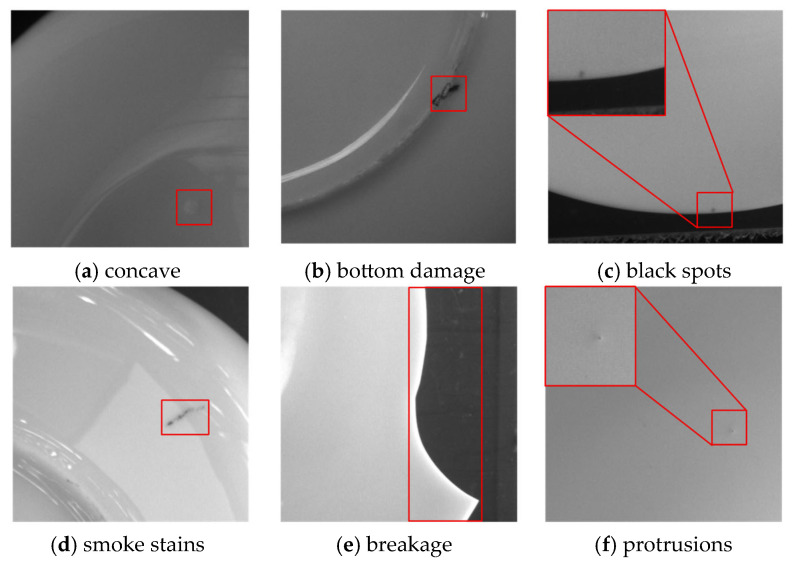
Different defects.

**Figure 7:**
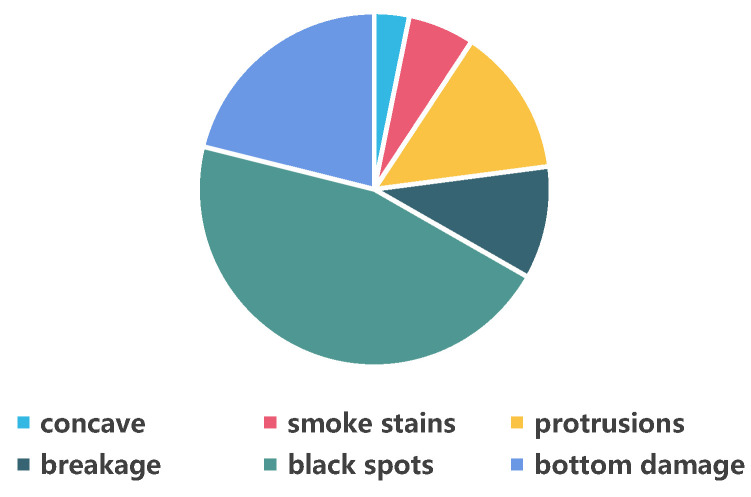
Proportion of Different Defects.

The team visited a ceramic manufacturing plant and collected 1067 defect images, including 4015 defects. The original image pixel size was 3840 × 2748. To make it suitable for the input size of the target detection model and avoid information loss, this section adopts the sliding window cropping strategy to divide the image. As shown in Figure 5, the high-resolution image is divided into 7 parts along the horizontal direction and 5 parts along the vertical direction. The adjacent sub-images have approximately 25% overlapping areas. Through this processing, each original high-resolution image can be divided into 35 sub-images of 640 × 640, including sub-images of different types of defects and normal defect-free images. The defect sub-images were made into the target detection training dataset, which includes 6 types of defects. Their shapes and proportions are shown in Figure 6 and Figure 7. Among them, the proportion of concave was 3.2%, bottom damage was 21.1%, black spots was 45.6%, smoke stains was 6.1%, breakage was 10.3%, and protrusions was 13.6%. Considering our goal of achieving data augmentation for rare ceramic defects and the current lack of high-quality public datasets on ceramics, this paper conducts experimental validation solely on this dataset.

Analysis shows that there is a class imbalance problem in the dataset. The proportions of “depressions” and “smoke stains” are relatively small, which will cause the trained model to have prediction biases and reduce the recognition ability for “depressions” and “smoke stains” defects. “Depressions” are surface flaws on ceramics that occur due to uneven contraction during high-temperature firing and cooling processes, and they appear on the front side of the ceramics; “smoke stains” are abnormal color spots attached to the bottom of the ceramics due to smoke exposure at the bottom, and they appear on the reverse side of the ceramics. Therefore, in this experiment, 100 images of “depressions”, 150 images of “smoke stains”, and the corresponding number of defect-free images on both the front and reverse sides of the ceramics were selected as the adversarial training dataset to conduct adversarial network training, hoping to obtain a sufficient number of new images of depressions and smoke stains to alleviate the sample imbalance problem.

**Figure 8:**
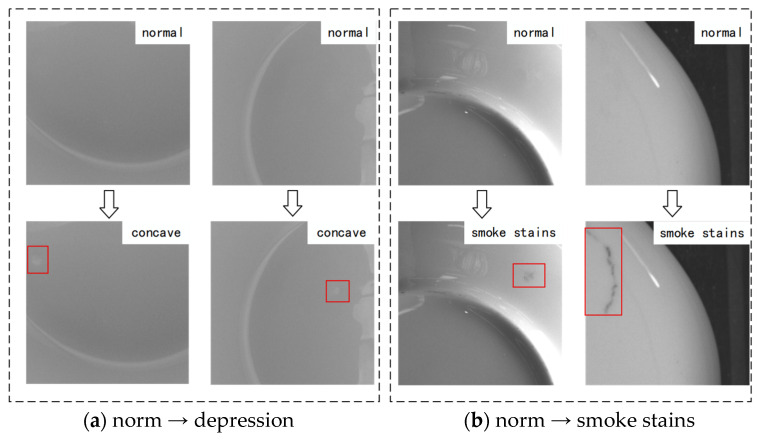
Synthetic target defect images generated from normal images by the TC-CycleGAN.

A total of two experiments were conducted in this section. In the first experiment, normal images and concave images were used for training. The trained model can achieve bidirectional conversion between normal images and concave images, and the experimental results are shown in Figure 8a. In the second experiment, smoke stains images and normal images were used, enabling the model to perform mutual conversion between normal and smoke stains images. The experimental results are shown in Figure 8b. A close observation of Figure 8 reveals that the generated concave and smoke-stained defect images have achieved a highly realistic, almost indistinguishable effect, which aligns with expectations.

### 3.3. Quantitative Analysis of Image Quality Enhancement

In this section, FID and KID are used for quantitative analysis to evaluate the generation quality of the defect images. FID removes the output layer of the InceptionV3 model and uses it as a feature extractor to extract the feature vectors of the real images and the generated images, and calculates the Fréchet distance of the feature vectors to measure the similarity between the generated images and the real images. The smaller the FID value is, the higher the quality of the generated images is. It can well evaluate the quality of the generated images, which is consistent with human visual judgment, and has a low computational complexity, and is widely applied. Its formula is shown in Equation (4), where x and y represent the real image and the generated image in the target domain, respectively; $\mu_{x}$ and $\mu_{y}$ represent the mean vector of the real image and the generated image, respectively. $\sigma_{x}$ and $\sigma_{y}$ represent the covariance matrices of the features of the real image and the generated image.

(4) $$\begin{array}{c} \mathrm{FID}(x,y)=∥\mu_{x}-\mu_{y}∥+\mathrm{Tr}\left(\sigma_{x}+\sigma_{y}-2{\left(\sigma_{x}\sigma_{y}\right)}^{1/2}\right) \end{array}$$

Similarly, KID removes the output layer of Inception-v3 to extract feature vectors, and then measures the quality of the generated image by calculating the maximum difference between the features of the real image and the generated image in the Reproducing Kernel Hilbert Space (RKHS). Unlike FID which assumes that the features follow a Gaussian distribution, KID uses the MMD with a polynomial kernel without any distribution assumption. Its estimator is unbiased and converges faster. KID performs well on small-scale datasets. The smaller the KID value, the closer the two distributions are, and the higher the quality of the generated image. The calculation is shown in Equations (5) and (6), where $x_{i}$ and $y_{i}$ represent the feature vectors of the real image and the generated image, respectively. $k\left(x,y\right)$ represents the polynomial kernel function p; n and m represent the number of real images and generated images respectively.

(5) $$k\left(x,y\right)={\left(\frac{1}{d}\langle x,y\rangle +1\right)}^{3},\;d=2048$$

(6) $$\begin{array}{rr} KID\left(X,Y\right) & =\frac{1}{n\left(n-1\right)}\sum_{i\neq j}\;k\left(x_{i},x_{j}\right)+\frac{1}{m\left(m-1\right)}\sum k\left(y_{i},y_{j}\right)-\frac{2}{nm}\sum_{i=1}^{n}\sum_{j=1}^{m}k\left(x_{i},y_{j}\right) \end{array}$$

**Figure 9:**
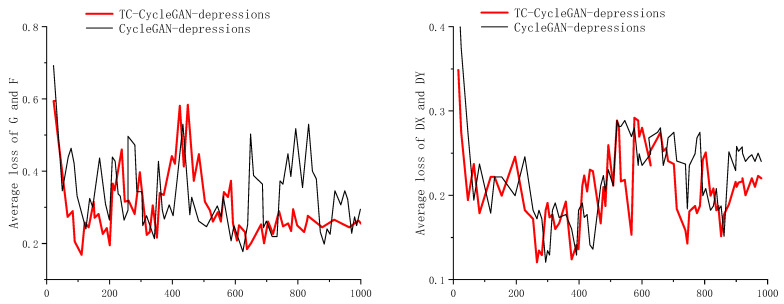
The generator and discriminator loss functions of CycleGAN and TC-CycleGAN.

This section first conducts ablation experiments to verify the significance of the optimizations made. During the experiments, the generator and discriminator are gradually optimized. The experimental results are shown in Table 1. It is noteworthy that a strict separation was maintained between the training dataset and the test dataset. All evaluation metrics were obtained from testing on images that were completely unseen by the model during training [25,26]. The variations in the generator and discriminator loss function values during training are shown in Figure 9. However, it is worth noting that in the training process of adversarial generative networks, when the generator’s performance improves and its loss decreases, the discriminator struggle to distinguish between real and generated samples, leading to an increase in the discriminator’s loss value. Conversely, when the discriminator’s performance improves, the generator’s loss value may also rise. This indicates that we cannot directly determine the specific performance of the model based solely on the magnitude of the loss function values.

However, evaluation metrics can provide a quantitative analysis. In Table 1, Table 2 and Table 3, the arrows (↓ and ↑) indicate the direction of the measurement indicators, while the symbol (√) indicates whether optimization measures have been taken. From Table 1, it can be seen that the optimized generator reduces the concave FID and KID by 63 and 9.6, respectively, and the smoke stains FID and KID by 120 and 12.1 respectively; the optimized discriminator reduces the concave FID and KID by 17 and 5.4 respectively, and the smoke stains FID and KID by 68 and 6.3 respectively; compared with the original CycleGAN network, the defects generated by the algorithm in this paper have a reduction of 88 and 13.5 in concave FID and KID, and a reduction of 137 and 13.3 in smoke stains FID and KID. These experiments fully prove that the optimization method proposed in this paper can effectively improve the quality of generated images. The concave FID has decreased by 48% and KID by 73%; the smoke stains FID has decreased by 57% and KID by 63%. The aforementioned experiments fully demonstrate the effectiveness of the two proposed improvement methods in the task of augmenting defects in daily use ceramics. The channel compression and squeeze-and-excitation attention mechanism in the TC-UNet generator enhance the model’s focus on key features and spatial information of ceramic defects. The DehazeFormer module significantly improves the model’s ability to fuse multi-scale features, thereby better accommodating the substantial variation in defect sizes in ceramics and contributing to the generation of more realistic defect images. The ContraNorm module in the TC-PatchGAN discriminator helps prevent the discriminator from collapsing during training and reduces its sensitivity to highlights on ceramic surfaces. Additionally, the patch-based discrimination mechanism helps preserve detailed texture information of the ceramics. Both the TC-UNet generator and TC-PatchGAN are well-suited for the task of augmenting defects in daily use ceramics, and their synergistic operation can significantly enhance the realism of the generated images.

Secondly, in order to further verify the superior performance of the model in this paper, comparative experiments were conducted with different adversarial networks. The experimental results are shown in Table 2. From the table, it can be seen that the image quality generated by the method in this paper is much higher than that of other adversarial networks, and this method meets the training requirements for ceramic defect detection.

### 3.4. Defect Detection Enhancement Experiment

Although TC-Cyclagan has achieved commendable results, it must be acknowledged that its reliance on generating flaws from existing data essentially imitates the original dataset, leading to issues such as unnatural data distribution and poor generalization. To further verify the effectiveness of the data augmentation method based on CycleGAN in improving the performance of object detection, this study trained the YOLOv8 model on the original dataset, the traditional augmented dataset, the CycleGAN augmented dataset, and the optimized CycleGAN augmented dataset, and compared and analyzed the final performance. At the same time, all three image augmentation methods retained a portion of the training set for object detection of the original images during the use process, and did not participate in the augmentation operation. This measure can ensure that the verification images are completely derived from the real distribution of defect images, thereby enabling a more objective evaluation of the model’s generalization performance in real scenarios and avoiding overestimation of the model’s actual application effect.

**Figure 10:**
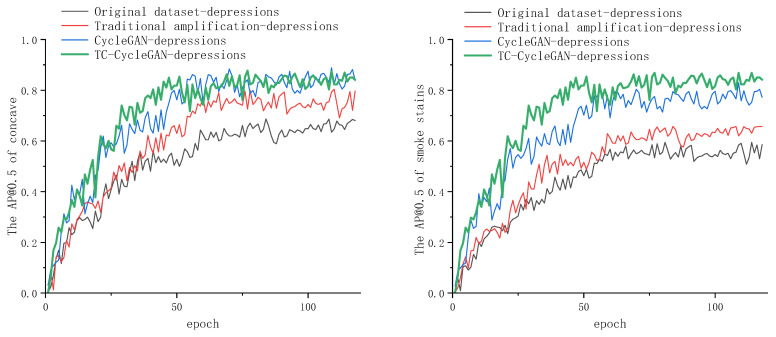
The AP curve of concave and smoke stains.

The experimental results are shown in Table 3 and Figure 10. AP@0.5 represents the average precision of a certain category when the IoU threshold is 0.5, while mAP@0.5 represents the average precision value of all categories at the 0.5 threshold. These three amplification methods exclusively amplify the rare defects (dents and smoke stains) without targeting other types of defects. Consequently, the indicators for dents and smoke stains are highlighted in bold. From the table, it can be seen that all three amplification methods can alleviate the problem of sample imbalance to some extent. However, the method TC-CycleGAN proposed in this paper achieved the best results. TC-CycleGAN can better adapt to the characteristics of daily use ceramics, such as feature blurring caused by highlights and the significant size variations among different defects. Compared with traditional amplification and CycleGAN amplification, the accuracy of concave increased by 6.5% and 1.2%, respectively, and the accuracy of smoke stains increased by 21% and 3.9% respectively. Figure 10 further demonstrates the extent to which the TC-CycleGAN-augmented dataset improves detector performance. Both graphs show that the detector trained with the TC-CycleGAN-augmented dataset achieves a faster increase in AP@0.5 and reaches a higher value, with the improvement being particularly evident for smoke stains defects.

The experimental results further verify that the data amplification method proposed in this paper can effectively alleviate the problem of model performance decline caused by sample imbalance, significantly improve the model’s detection ability for insufficiently sampled defects, and increase the defect detection accuracy.

## 4. Conclusions

In view of the current image augmentation techniques’ limitations in the task of expanding rare ceramic defects, such as uneven brightness and insufficient small-sized defect features, it is difficult to generate high-quality defect images, and thus shows limited improvements on defect detection accuracy. It is unable to effectively solve the problems faced by deep learning-based ceramic defect detection technology, such as the shortage of rare defect samples and the imbalance of categories. This paper proposes a ceramic rare defect image augmentation method based on TC-CycleGAN. The generator adopts TC-UNet, and by introducing the scSE attention mechanism and the DehazeFormer module, it enhances the model’s ability to extract and reconstruct the subtle defect features on the ceramic surface; the discriminator adopts PC-PatchGAN and replaces the original BatchNorm module with the ContraNorm module, effectively alleviating the overfitting problem of the discriminator to simple features, improving its sensitivity to feature representation, and enhancing the diversity of the generated images. In the quantitative analysis experiment of image quality enhancement, the method proposed in this paper significantly improved the visual quality and distribution authenticity of the generated defective images. The FID and KID indicators for the concave generation images reached 92 and 4.8, respectively, while for the smoky images, the FID and KID were 103 and 7.9, respectively, both superior to CycleGAN and some classic GAN networks. Among them, the FID and KID of the concave images decreased by 49% and 73%, respectively, and those of the smoky images decreased by 57% and 63%, respectively, indicating that the images generated by the improved method in this paper are closer to the real defective images in terms of visual quality and data distribution. The further defect detection improvement experiment results show that using the data set expanded by this method for training can effectively alleviate the problem of class imbalance and significantly improve the recognition ability of the detection model for rare defects. Among them, the detection accuracy of concave defects increased by 1.2% and that of smoky defects increased by 3.9%, verifying the effectiveness and application potential of the proposed method in practical detection tasks.

However, the current research has yet to integrate the dataset construction methods and defect recognition algorithms suitable for ceramic defect detection with related hardware systems—including image acquisition solutions and automated sorting methods for daily use ceramics—thus preventing large-scale application in ceramic manufacturing enterprises. In the future, we will conduct in-depth research on these aspects to promote the practical implementation of this technology in real production environments, thereby supporting the intelligent transformation and upgrading of ceramic enterprises.

## Figures and Tables

**Table 1:** Ablation experiment.

CycleGAN		Concave		Smoke Stains	
Generator	Discriminator	FID ↓	KID ↓	FID ↓	KID ↓
√		180	18.3	240	21.2
√		117	8.7	120	9.1
	√	163	12.9	172	14.9
√	√	92	4.8	103	7.9

**Table 2:** Contrast experiment.

Model	Concave		Smoke Stains	
	FID ↓	KID ↓	FID ↓	KID ↓
VAE	436	71.6	480	79.6
AE	259	29	310	31.2
Defect-Gen	193	19.1	198	19.3
DFMGAN	135	9.9	170	12.8
StyleGAN v2	102	7.5	115	8.4
CycleGAN	180	18.3	240	21.2
Ours	92	4.8	103	7.9

**Table 3:** The impact of different amplification methods on the performance of the detector.

Amplification Method	AP@0.5 ↑						mAP@0.5 ↑
	Concave	Smoke Stains	Bottom Damage	Black Spots	Breakage	Protrusions	
Original dataset	**68.7%**	**59.4%**	95.7%	71.2%	94.0%	79.3%	78.1%
Traditional amplification	**80.9%**	**65.7%**	95.8%	71.5%	93.4%	81.0%	81.3%
CycleGAN expansion	**86.2%**	**82.8%**	96.0%	69.1%	94.2%	81.6%	84.9%
Ours	**87.4%**	**86.7%**	95.3%	71.7%	93.9%	81.2%	86.03%

## Data Availability

The original contributions presented in this study are included in the article. Further inquiries can be directed to the corresponding author.

## References

[1] Hondo T., Yasuda K., Wakai F., Tanaka S. (2018). Influence of binder layer of spray-dried granules on occurrence and evolution of coarse defects in alumina ceramics during sintering. J. Eur. Ceram. Soc..

[2] Sun H., Li J., Zhu X. (2025). A Novel Expandable Borderline Smote Oversampling Method for Class Imbalance Problem. IEEE Trans. Knowl. Data Eng..

[3] Shorten C., Khoshgoftaar T.M. (2019). A survey on image data augmentation for deep learning. J. Big Data.

[4] Park S., Kim J., Wang S., Kim J. (2025). Effectiveness of Image Augmentation Techniques on Non-Protective Personal Equipment Detection Using YOLOv8. Appl. Sci..

[5] Riles A. (2008). The anti-network: Private global governance, legal knowledge, and the legitimacy of the state. Am. J. Comp. Law.

[6] Ho J., Jain A., Abbeel P. (2020). Denoising diffusion probabilistic models. Adv. Neural Inf. Process. Syst..

[7] Hu H.X., Cao C., Hu Q., Zhang Y., Lin Z.-Z. (2023). A real-time bearing fault diagnosis model based on siamese convolutional autoencoder in industrial internet of things. IEEE Internet Things J..

[8] He S., Zhou F., Tan X., Hu G., Ruan J., He S. (2025). Research on Mechanical Fault Diagnosis Method of Isolation Switch Based on Variational Autoencoder. Processes.

[9] Decourt C., Duong L. (2020). Semi-supervised generative adversarial networks for the segmentation of the left ventricle in pediatric MRI. Comput. Biol. Med..

[10] Chen W., Li Y., Zhao Z. (2022). Transmission line vibration damper detection using multi-granularity conditional generative adversarial nets based on uav inspection images. Sensors.

[11] Ou H., An J., Wang X.-A., Xiong J., Chen X., Wang Q. A StyleGAN3-Based Data Augmentation Method for Ceramic Defect Detection. Proceedings of the 2023 IEEE 6th International Conference on Industrial Cyber-Physical Systems (ICPS).

[12] Yang B., Liu Z., Duan G., Tan J. (2021). Mask2Defect: A prior knowledge-based data augmentation method for metal surface defect inspection. IEEE Trans. Ind. Inform..

[13] Lv N., Ma H., Chen C., Pei Q., Zhou Y., Xiao F., Li J. (2021). Remote sensing data augmentation through adversarial training. IEEE J. Sel. Top. Appl. Earth Obs. Remote Sens..

[14] Wang Y., Hu W., Wen L., Gao L. (2023). A new foreground-perception cycle-consistent adversarial network for surface defect detection with limited high-noise samples. IEEE Trans. Ind. Inform..

[15] Zhou S., Jia W., Diao H., Geng X., Wu Y., Wang M., Wang Y., Xu H., Lu Y., Wu Z. (2025). A CycleGAN-Pix2pix framework for multi-objective 3D urban morphology optimization: Enhancing thermal performance in high-density areas. Sustain. Cities Soc..

[16] Xia Y., Han S.W., Kwon H.J. (2023). Image generation and recognition for railway surface defect detection. Sensors.

[17] Chen K., Li H., Li C., Zhao X., Wu S., Duan Y., Wang J. (2022). An automatic defect detection system for petrochemical pipeline based on cycle-gan and yolo v5. Sensors.

[18] Alam L., Kehtarnavaz N. (2023). Generating defective epoxy drop images for die attachment in integrated circuit manufacturing via enhanced loss function cyclegan. Sensors.

[19] Roy A.G., Navab N., Wachinger C. Concurrent Spatial and Channel ‘Squeeze & Excitation’ in Fully Convolutional Networks. Proceedings of the 21st International Conference on Medical Image Computing and Computer-Assisted Intervention (MICCAI 2018).

[20] Song Y., He Z., Qian H., Du X. (2023). Vision transformers for single image dehazing. IEEE Trans. Image Process..

[21] Zhang S., Wang H., Wang L. A Sensitive Image Generation Method Based on Improved PatchGAN. Proceedings of the 2023 12th International Conference of Information and Communication Technology (ICTech).

[22] Nguyen T., Yoo M. PatchGAN-Based Depth Completion in Autonomous Vehicle. Proceedings of the 2022 International Conference on Information Networking (ICOIN).

[23] Zhao Y., Bai L., Huang X. Fidnet: Lidar Point Cloud Semantic Segmentation with Fully Interpolation Decoding. Proceedings of the 2021 IEEE/RSJ International Conference on Intelligent Robots and Systems (IROS).

[24] Bińkowski M., Sutherland D.J., Arbel M., Gretton A. (2018). Demystifying mmd gans. arXiv.

[25] Armaghani D.J., Asteris P.G. (2021). A comparative study of ANN and ANFIS models for the prediction of cement-based mortar materials compressive strength. Neural Comput. Appl..

[26] Asteris P.G., Skentou A.D., Bardhan A., Samui P., Pilakoutas K. (2021). Predicting concrete compressive strength using hybrid ensembling of surrogate machine learning models. Cem. Concr. Res..

